# A Pilot Study of Exploring miRNA–Protein Interaction Networks in Pancreatic Ductal Adenocarcinoma Patients: Implications for Diagnosis and Prognosis

**DOI:** 10.3390/diagnostics15192479

**Published:** 2025-09-27

**Authors:** Sena Şen, Merve Çiğdem Özgel, Şeref Buğra Tunçer, Hamza Uğur Bozbey, Senem Karabulut, Didem Taştekin

**Affiliations:** 1 Department of Basic Oncology, Oncology Institute, Istanbul University, 34093 Istanbul, Türkiye; sena.sen@istanbul.edu.tr (S.Ş.); mervecigdemozgel@ogr.iu.edu.tr (M.Ç.Ö.); seref.tuncer@istanbul.edu.tr (Ş.B.T.); 2 Department of Basic Oncology, Institute of Health Sciences, Istanbul University, 34126 Istanbul, Türkiye; 3 Department of Clinic Oncology, Oncology Institute, Istanbul University, 34093 Istanbul, Türkiye; ugurbozbey@yahoo.com (H.U.B.); senem.karabulut@istanbul.edu.tr (S.K.)

**Keywords:** biomarker, expression, in silico analysis, miRNA, pancreatic ductal adenocarcinoma

## Abstract

**Background:** Pancreatic ductal adenocarcinoma (PDAC) is one of the most lethal malignancies for which there are few effective biomarkers for diagnosis, prognosis, and treatment monitoring. Given the paucity of data in the literature, this study aimed to evaluate the biomarker potential of selected miRNAs (miR-222-3p, miR-3154, miR-3945, miR-4534, and miR-4742) and their protein targets in the context of PDAC. **Methods:** The expression levels of miRNA candidates were quantified by real-time quantitative PCR in lymphocyte samples from 46 PDAC patients and 50 healthy controls. In silico analyses were performed to identify potential target genes and proteins. ELISA was used to measure protein expression in both groups. Statistical analyses included ROC curve analysis, linear regression, and correlation analyses. In addition, correlations between miRNA/protein expression and clinicopathologic characteristics, including survival, were investigated. **Results:** miR-222-3p and miR-3154 were significantly downregulated in PDAC patients compared to controls (*p* < 0.001). Among the dual miRNA combinations, miR-222-3p and miR-4534 showed the highest discriminatory power (AUC = 0.629, *p* = 0.022). The miR-222-3p expression was significantly increased in patients with a history of alcohol consumption (*p* = 0.02). Significant correlations were observed between miR-3154 expression and T-stage (*p* = 0.01) and between perineural invasion and miR-222-3p levels (*p* = 0.02). Survival analysis showed that high miR-3945 expression was significantly associated with shorter overall survival (*p* = 0.001). Elevated levels of ESR1, HCFC1, and EPC1 were significantly associated with lymphatic invasion (*p* < 0.05), while high KCNA1 expression correlated with shorter survival (*p* = 0.006), indicating its potential as a negative prognostic biomarker. Linear regression analysis revealed a significant positive correlation between miR-3945 and KCNA1 expression (β = 0.259, *p* = 0.038), indicating a possible regulatory interaction. A borderline correlation was also found between miR-4742 and EPC1 expression (*p* = 0.055). **Conclusions:** This study identifies several miRNAs and associated proteins with diagnostic and prognostic significance in PDAC. The results emphasize the clinical relevance of integrating multi-layered analyses of miRNA–protein interactions. The observed associations highlight the role of these molecular markers in tumor progression and patient survival and offer promising opportunities for future research and clinical application in precision oncology.

## 1. Introduction

Pancreatic cancer comprises several histologic subtypes, including pancreatic ductal adenocarcinoma (PDAC), pancreatic neuroendocrine tumors, acinar cell carcinomas, and mucinous cystic neoplasms [1]. Among them, PDAC accounts for more than 90% of all pancreatic malignancies and is characterized by its aggressive biology and poor prognosis. It develops through the interaction of genetic and epigenetic mechanisms [2]. Epigenetic modifications (such as DNA methylation, histone modifications, and mechanisms mediated by non-coding RNAs) are known to influence gene expression in a stable yet reversible manner [3]. According to GLOBOCAN 2018 data, PDAC ranks 14th worldwide in incidence but 7th in cancer-related mortality due to its poor prognosis. The incidence and mortality rates of PDAC are similar in men and women [4]. Both hereditary and environmental factors are associated with the development of the disease. Risk factors such as smoking, alcohol consumption, obesity, diabetes mellitus, a history of chronic pancreatitis, a family history, and hereditary cancer syndromes (including Hereditary Breast and Ovarian Cancer and von Hippel–Lindau, Peutz–Jeghers, and Lynch syndromes) have been identified in the development of PDAC [5]. While environmental factors are known to influence gene expression through epigenetic mechanisms, growing attention is being directed toward the regulatory role of non-coding RNAs in this process. microRNAs (miRNAs) are small (19–24 nucleotides), regulatory, single-stranded non-coding RNA molecules [6]. They are involved in various biological processes such as cell proliferation, apoptosis, and differentiation [7]. Depending on the binding of miRNAs to their target sequences, gene expression can be suppressed or enhanced. In addition, miRNAs also act as signaling molecules that influence the behavior of various other signaling components. In PDAC, various miRNAs act as oncogenes or tumor suppressors, playing crucial roles in tumor biology [8]. Oncogenic miRNAs (e.g., miR-10a, -21, -132/-212, -155, -194/-200 family, -197, -221, -224, -486) promote proliferation, invasion, metastasis, and chemoresistance, whereas tumor-suppressive miRNAs (e.g., miR-301a, -320c, -491-5p, let-7 family, miR-34 family, miR-143, -148b, -150, -200, -375, -548d) inhibit tumor progression by inducing apoptosis and limiting growth and spread [9,10]. Circulating and exosomal miRNAs, due to their high stability, have emerged as promising non-invasive biomarkers. In particular, serum/plasma levels of miR-21, -18a, -155, -196a, and -221 have been associated with PDAC diagnosis, prognosis, and treatment response, highlighting their potential as clinical tools [11]. Therefore, the detection of miRNAs in body fluids has attracted great interest as they could be used as biomarkers [12]. One of the main factors for the poor prognosis of PDAC is its late diagnosis and its biological resistance to treatment [13]. In this context, the potential of miRNAs to serve as both diagnostic and therapeutic targets is increasingly being investigated.

In a comprehensive study conducted by O’Neill et al. in 2023 [14], miRNA expression levels were compared using the Affymetrix GeneChip miRNA 4.0 platform in samples from human pancreatic tumors, adjacent normal tissues and patient-derived xenograft (PDX) models. Through these analyses, several miRNAs that were previously uncharacterized in the context of PDAC were identified as differentially expressed for the first time. In particular, lesser-known miRNAs such as miR-4534, miR-3154, miR-4742, miR-222-3p, and miR-3945 showed significant expression differences. These changes in miRNA expression were not only observed at the transcriptomic level, but also confirmed by proteomic analyses showing that their target proteins are involved in various biological processes, especially aldehyde metabolism and membrane transport. The reported proteins were found to be significantly associated with miRNA-mediated regulation, and in this context, miRNA–protein interactions have been proposed as potential therapeutic targets.

In line with the above literature, this study aimed to determine the gene and protein expression levels of five newly identified miRNAs—miR-4534, miR-3154, miR-4742, miR-222-3p, and miR-3945—in blood samples from individuals diagnosed with PDAC and healthy controls. These miRNAs were evaluated for their potential utility as diagnostic markers for disease detection, prognostic markers for disease progression, and predictive biomarkers for assessing response to targeted, cytotoxic, and anti-metastatic therapies. This study investigated not only the expression levels of the miRNA candidates at the gene level, but also the protein expression of their prospective target gene products. This approach was designed to clarify how miRNAs regulate gene expression at the post-transcriptional level and how this regulation is reflected at the post-translational level in the form of protein synthesis. This comprehensive strategy was developed to provide a multi-layered understanding of the functional role of miRNAs in PDAC. Both in silico bioinformatic analyses and experimental studies at the translational level were used to track the effects of miRNAs on cellular processes to the final molecular outcomes. These analyses enabled a detailed assessment of the diagnostic accuracy, prognostic significance, and impact on survival of the miRNA candidates and their associated target genes/proteins in the context of PDAC. The data obtained should provide new perspectives for basic research.

## 2. Materials and Methods

This study conformed to the tenets of the 75th Declaration of Helsinki (2024) [15] and was approved by the institution’s ethics committee. Informed consent was obtained from all participants prior to the collection and analysis of samples and clinical data. All PDAC patients and healthy individuals who voluntarily participated in the study were provided with detailed information about the scope of the research by their attending clinicians. Power analysis was performed using the Power and Sample Size program, with a Type I error set at 0.05 and a test power of 0.80. It was determined that a minimum of 16 subjects per group was required to detect a 30.00-unit difference between group means.

The inclusion criteria for the patient group were as follows: a histopathologically confirmed diagnosis of PDAC, no prior systemic treatment (such as chemotherapy, radiotherapy, immunotherapy, etc.), age between 18 and 80 years, and provision of written informed consent. For the control group, individuals were required to have no personal or first-degree family history of malignant or benign tumors. Exclusion criteria included prior oncological treatment, diagnosis of another concurrent primary malignancy, histopathological subtypes other than pancreatic adenocarcinoma, presence of severe systemic illness, or active infection. As a result of histopathological evaluation, three patients diagnosed with neuroendocrine tumors and one patient with a mucinous subtype were excluded from the study. This study was subsequently continued with 46 patients. Control group samples were obtained from 50 healthy volunteers selected based on age- and sex-matching. Randomization was not performed.

### 2.1. Sample Collecting

Prior to treatment, peripheral blood samples (8–10 mL) from patients with PDAC and healthy volunteers were collected in serum separation tubes (dry tubes) and delivered to the laboratory. Once there, the samples were centrifuged at 1950 rpm for 30 min. After centrifugation, the yellow-colored serum phase that had formed at the top was carefully separated to avoid contamination, transferred to 1 mL aliquots in cryotubes, and stored at −20 °C. In addition, 10 mL samples of peripheral blood from patients diagnosed with PDAC and healthy controls were collected in tubes containing ethylenediaminetetraacetic acid (EDTA). The collected samples were carefully transferred to Falcon tubes containing Ficoll solution and centrifuged at 1500 rpm for 30 min. After centrifugation, the mononuclear cell layer (lymphocytes, monocytes, etc.) that had accumulated at the interface was carefully aspirated and transferred to new tubes. This cell suspension was then centrifuged again at 1950 rpm for 10 min. The supernatant was discarded, and 4 mL of phosphate-buffered saline (PBS) was added to the resulting pellet. The final cell suspension was aliquoted into four separate cryovials (1 mL each). The samples were stored at −80 °C in the freezer until the experimental procedures were performed.

### 2.2. Isolation of miRNA

Total RNA, including miRNA, was isolated from peripheral blood lymphocyte samples of PDAC patients using the NucleoSpin^®^ miRNA kit (Lot No. 2405-001) (Macherey-Nagel, Düren, Germany) according to the manufacturer’s instructions. First, the cell or tissue samples were completely homogenized in 300 µL of MP buffer. Subsequently, 300 µL of MPL buffer was added to complete the lysis process, and the mixture was shaken thoroughly. To precipitate the nucleic acids, 600 µL of 96–100% ethanol was added to the lysate and mixed again. The resulting mixture was then transferred to the primary NucleoSpin^®^ miRNA column to allow binding of the RNA to the silica membrane and centrifuged at 11,000× *g* for 30 s. The flow-through was collected in a new tube, transferred to the secondary column, and centrifuged under the same conditions. The primary column was then placed back into its original collection tube. For the washing steps, 300 µL of MW1 buffer was added to the primary column and centrifuged, followed by the addition of 500 µL of MW2 buffer and subsequent centrifugation. The column was then transferred to an empty tube and centrifuged for one minute to ensure complete drying. Similarly, the secondary column was washed with 300 µL of MW1 buffer and 500 µL of MW2 buffer and then transferred to an empty tube and centrifuged to dry. During the elution step, 30–100 µL of REB buffer was added to both columns, followed by a one-minute centrifugation to elute the RNA. The isolated RNA samples were stored at −70 °C for long-term storage and −20 °C for short-term use. After isolation, the RNA samples were quantified and analyzed for purity using a Nanodrop 2000 spectrophotometer (Thermo Fisher Scientific, Waltham, MA, USA). The cDNA synthesis was then performed.

### 2.3. Quantitative Analysis of miRNA Expression

The A.B.T.™ Single miRNA qPCR Assay Kit (Lot No. W2C0223-Q10) (Ankara, Türkiye) was used to determine the expression levels of miRNAs from RNA samples. This SYBR-Green master kit consists of two main components: the miR-cDNA Synthesis Kit and the miR-qPCR MasterMix. All procedures were performed under aseptic conditions and in accordance with the manufacturer’s instructions. The conversion of cellular RNA-derived miRNAs into cDNA was performed. A total reaction volume of 6 μL was prepared using 4 μL miR-cDNA synthesis kit and 2 μL miRNA template. Primer sequences are summarized in Table 1. All reagents were thawed at room temperature and then kept on ice. The reaction mixture was homogenized and briefly centrifuged to prevent precipitation. The synthesized miRNA-cDNA samples were either immediately subjected to real-time PCR analysis or stored at −20 °C for further use. For qPCR reactions, a final volume of 10 μL was prepared with 8 μL of miR-qPCR MasterMix and 2 μL of cDNA template. The reaction mixtures were thoroughly mixed by pipetting and briefly centrifuged to ensure homogenization. The prepared reactions were then added to a 96-well plate and loaded into a real-time PCR system. The PCR protocol consisted of an initial denaturation step at 95 °C for 10 min (1 cycle), followed by 30 to 40 cycles of denaturation at 95 °C for 10 s and annealing/detection at 60 °C for 25 s. Subsequent melting curve analysis was performed from 60 °C to 95 °C with 2–5 s steps at each step to evaluate the specificity of amplification. Ct values (cycle threshold) were normalized to the U6 reference gene, and targeted miRNA expression levels were calculated using the 2^−ΔΔCt^ method based on two independent measurements [16].

### 2.4. In Silico Prediction and Network-Based Functional Analysis of miRNA Target Genes

The potential target genes of miR-4534, miR-3154, miR-4742, miR-222-3p, and miR-3945 were predicted using the miRDB database [17]. For each miRNA, the predicted target genes were ranked according to the miRDB target score, and only those with a score ≥ 80 were included in further analyses, as this threshold is generally accepted as an indication of a high reliability of predictions in miRDB. The identified target genes were then imported into Cytoscape software (v3.10.0, Institute for Systems Biology, Seattle, WA, USA) to create a gene–miRNA interaction network. To identify hub genes within the network topology, the CytoHubba plugin available on the Cytoscape platform was used. In this analysis, the Maximal Clique Centrality (MCC) algorithm was used to determine the most critical genes within the network. Genes with high MCC scores were considered as candidates with potential central biological importance.

### 2.5. Determination of Target Protein Levels by Enzyme-Linked Immunosorbent Assay (ELISA)

Quantitative analysis of ESR1 (Human Estrogen Receptor Alpha, ESR1 ELISA Kit, E7871Hu), HCFC1 (Human Host Cell Factor C1, HCFC1 ELISA Kit, E0307Hu), KCNA1 (Human Voltage-Gated Potassium Channel Subunit Alpha-1, KCNA1 ELISA Kit, E1757Hu), EPC1 (Human Enhancer of Polycomb Homolog 1, EPC1 ELISA Kit, E1137529Hu), and CACNG3 (Human Voltage-Dependent Calcium Channel Subunit Gamma-3, CACNG3 ELISA Kit, E4572Hu) proteins in the serum samples of PDAC patients was performed using sandwich-format ELISA kits (BT Lab, Bioassay Technology Laboratory, Shanghai, China). The blood samples from patients and healthy individuals were allowed to clot at room temperature for 10–20 min and then centrifuged at 2000–3000 rpm for 20 min. The resulting supernatant (serum) was carefully collected and separated to avoid cell debris. Prior to analysis, all samples were brought to room temperature and homogenized. Samples were stored at −80 °C until analysis, and freeze–thaw cycles were avoided. All reagents were brought to room temperature and incubated for the recommended time before use. To prepare the standard curve, a standard solution with a concentration of 96 ng/mL was serially diluted (1:2) to obtain concentrations of 48, 24, 12, 6, and 3 ng/mL using the standard diluent provided by the manufacturer. The wash buffer was prepared by diluting the 25× concentrate with distilled water to obtain a 1× working solution. The 96-well ELISA plate was pre-coated with antibodies specific for the target proteins by the manufacturer. During the assay, 40 μL of the serum sample, 10 μL of the biotin-labeled antibody, and 50 μL of the streptavidin–HRP conjugate were added to each well. The mixture was homogenized, and the plate was sealed with an adhesive foil and incubated at 37 °C for 60 min. After incubation, the wells were washed five times with wash buffer. Subsequently, 50 μL of substrate A and 50 μL of substrate B were added to each well, and the plate was incubated for 10 min at 37 °C in the dark. The reaction was terminated by adding 50 μL of stop solution, and the color change from blue to yellow was observed. Absorbance measurements were performed within 10 min after the addition of the stop solution using a microplate reader set to 450 nm wavelength. The optical density (OD) values obtained were evaluated with respect to the standard curve. The standard curve was constructed by plotting the mean OD values for each standard concentration and fitted with the best-fitting regression model. Sample concentrations were calculated by interpolation from the standard curve. Inter-assay and intra-assay variability were assessed by calculating the coefficient of variation (CV%), which was less than 8% and 10%, respectively.

### 2.6. Statistical Analysis

Statistical analyses were performed using SPSS statistical software version 30.0 (SPSS Inc., Chicago, IL, USA) to assess the association between clinical parameters and expression status using chi-square tests (Pearson chi-square test, Yates’ continuity correction, and Fisher’s exact test). Categorical variables were expressed as n (%). The Pearson chi-square test was applied when the expected frequency met the conditions T ≥ 5 and *N* ≥ 40; the chi-square test with Yates’ continuity correction was used when T ≥ 5 but *N* < 40; and Fisher’s exact test was applied when T < 1, *N* < 40, or when zero units of observation were present. For categorical variables with more than two subgroups, if the expected frequencies in some cells were less than 5 and the assumptions of the classical chi-square test were not met, the Monte Carlo simulation method was used to obtain more reliable significance levels. A *p*-value ≤ 0.05 was considered statistically significant. Normality of the distribution of continuous variables, including age and expression levels of selected miRNAs and proteins, was assessed using the Kolmogorov–Smirnov test. The effects of age and gender on expression levels were analyzed using linear regression analysis. The expression levels of selected miRNAs and proteins between the healthy control group and the PDAC patients were compared using the Mann–Whitney U test. Receiver operating characteristic (ROC) curve analyses were performed to evaluate the diagnostic potential of selected miRNAs and proteins. A *p*-value < 0.05 was considered statistically significant. Based on the median cut-off values of selected miRNAs and proteins, patients were divided into low and high expression groups and further classified according to clinical parameters. Survival analysis was performed using the Kaplan–Meier method, and differences between survival curves were evaluated using the log-rank test. The relationships between survival outcomes and the independent variables were assessed using univariate and multivariate Cox proportional hazards regression analyses, and the results were visualized with a forest plot. Hazard ratios (HRs) with 95% confidence intervals (CIs) were reported for all Cox regression models. Linear regression analyses were performed to investigate the potential regulatory relationships between selected microRNAs and their predicted target proteins. Each miRNA–protein pair was evaluated separately, treating miRNA as the predictor variable and the corresponding protein expression level as the outcome variable. Analyses used the Enter method, and model assessment was based on the regression coefficient (β), coefficient of determination (R^2^), and statistical significance (*p*-value). A *p*-value below 0.05 was considered statistically significant. Only data entries without missing values were included in the analysis.

## 3. Results

### 3.1. Demographics and Clinicopathological Characteristics

The demographic and clinicopathologic characteristics of the 46 patients included in the study were evaluated and are listed in Table 2. The age of the patients ranged from 34 to 90 years, with a mean age of 53.5 years. The gender distribution was almost equal, with 47.8% of patients identifying as female and 52.2% as male. Healthy volunteers were age- and sex-matched with patients. Smoking was reported in the medical history in 56.5% of cases, while 21.7% reported alcohol consumption. Demographic and lifestyle data were also analyzed for the control group, which consisted of 50 healthy individuals. The age of the participants ranged from 26 to 71 years, with an average age of 45 years. The gender distribution was 48% female and 52% male. In terms of smoking, 72% of participants reported having smoked in the past, while 28% of participants did not smoke. In terms of alcohol consumption, 44% of participants reported consuming alcohol, while 56% reported abstinence.

A positive family history of malignancies was documented in 54.7% of patients. Diabetes mellitus was present in 43.5% of cases, and at least one concomitant disease was found in 73.9% of patients. In terms of anatomical localization, the head of the pancreas was the most frequently affected region (60.9%), followed by the body (21.7%) and the tail (17.4%). Histopathologic examination revealed that all cases were diagnosed as adenocarcinoma. In terms of tumor differentiation, 78.3% of the tumors were moderately differentiated, while 21.7% were poorly differentiated. Positive surgical margins were observed in 52.2% of cases. Lymphatic invasion was found in 82.6% of patients, and perineural invasion in 71.7%. According to the TNM classification, 37.9% of the tumors were classified as T2, 45.7% as T3, and 17.4% as T4. At initial diagnosis, the lymph nodes were affected in 76.1% of cases, and distant metastases were detected in 45.7% of cases. Based on the WHO staging system, the largest proportion of patients (45.7%) were classified as stage IV, followed by stage IIb (23.9%). Locally advanced disease was found in 80.4% of patients. In terms of treatment modalities, 58.7% of patients received chemotherapy and 39.1% received chemoradiotherapy, while one patient refused treatment due to personal choice. During the follow-up period, recurrence was detected in 15.2% of patients, and distant metastases in 28.3%. At the end of the follow-up period, 67.4% of patients had died, while 32.6% were still alive.

### 3.2. Expression Analyses of Selected miRNAs

**Figure 1:**
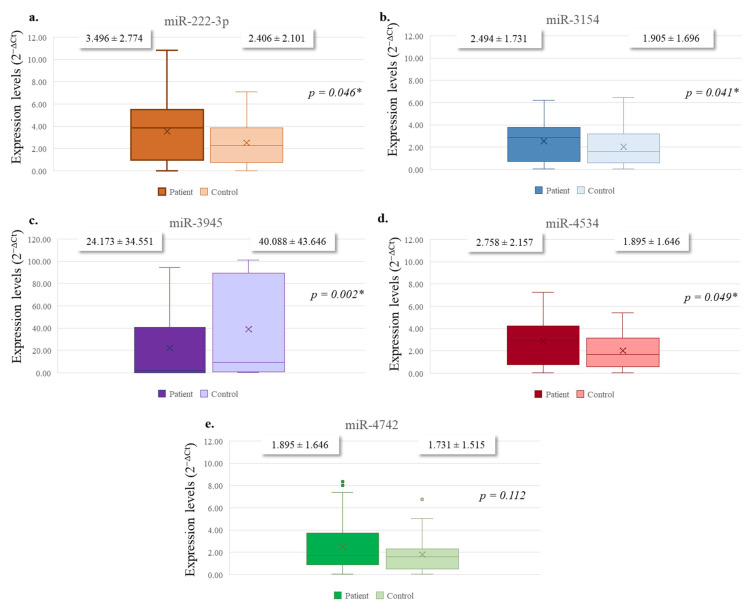
The distribution of lymphocyte levels of the selected miRNAs between PDAC patients and healthy individuals. (**a**) miR-222-3p, (**b**) miR-3154, (**c**) miR-3945, (**d**) miR-4534, and (**e**) miR-4742. Differences between the groups were analyzed using the Mann–Whitney U test. Bars represent mean values of expression, and error bars indicate standard deviation (mean ± SD). * *p* < 0.05 was considered statistically significant.

The expression of miR-222-3p (mean ± SD: 3.496 ± 2.774) and miR-3154 (mean ± SD: 2.494 ± 1.731) was significantly higher in patients than in healthy controls (mean ± SD: 2.406 ± 2.101, *p* = 0.046; mean ± SD: 1.905 ± 1.696, *p* = 0.041, respectively). A marked decrease was observed in miR-3945 expression in patients (mean ± SD: 24.173 ± 34.551) compared to healthy individuals (mean ± SD: 40.088 ± 43.646, *p* = 0.002). Similarly, miR-4534 levels were elevated in patients (mean ± SD: 2.758 ± 2.157) compared to controls (mean ± SD: 1.895 ± 1.646, *p* = 0.049). In contrast, no significant difference was detected in the expression levels of miR-4742-5p between the two groups (mean ± SD: 2.439 ± 2.244 vs. 1.731 ± 1.515, *p* = 0.112) (Figure 1).

**Figure 2:**
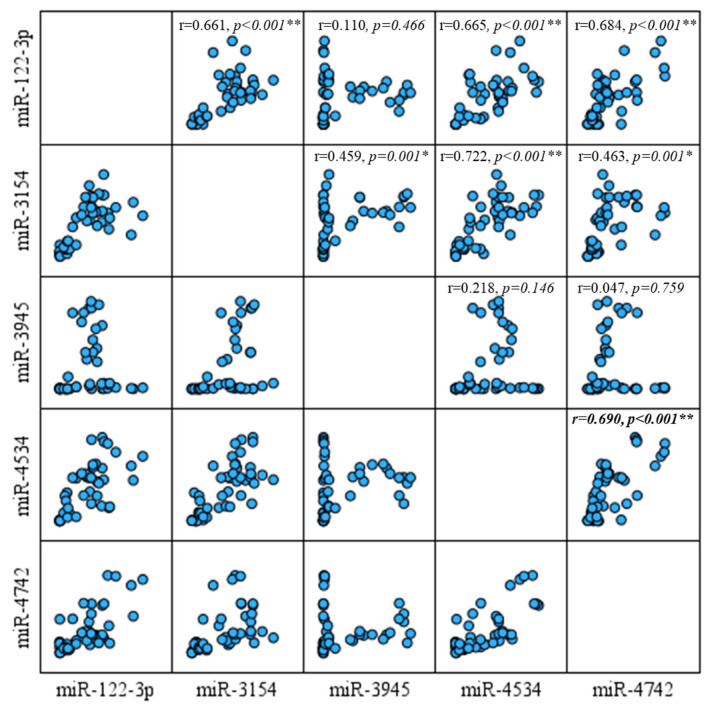
Scatter plot matrix demonstrating the pairwise correlations among expression levels of miR-222-3p, miR-3154, miR-3945, miR-4534, and miR-4742. The upper triangle of the matrix displays Pearson correlation coefficients (r) with corresponding *p*-values. Significant positive correlations were observed for each miRNA. * *p* < 0.05 and ** *p* < 0.001 were considered statistically significant.

Bivariate Pearson correlation analysis was performed to assess the associations between the expression levels of the selected miRNAs in the patient group and visualized through a scatter plot matrix (Figure 2). A strong and statistically significant positive correlation was found between miR-222-3p and miR-3154 (r = 0.661, *p* < 0.001), miR-4534 (r = 0.665, *p* < 0.001), and miR-4742 (r = 0.684, *p* < 0.001). Likewise, miR-3154 expression was positively correlated with miR-3945 (r = 0.459, *p* = 0.001), miR-4534 (r = 0.722, *p* < 0.001), and miR-4742 (r = 0.463, *p* = 0.001). A strong positive correlation was also observed between miR-4534 and miR-4742 (r = 0.690, *p* < 0.001). No statistically significant correlations were found between miR-3945 and miR-222-3p (r = 0.110, *p* = 0.466), miR-4534 (r = 0.218, *p* = 0.146), or miR-4742 (r = 0.047, *p* = 0.759).

#### 3.2.1. Biomarker Potentials of Selected miRNAs and Their Combinations

To evaluate the diagnostic performance of the candidate miRNAs in PDAC, ROC curve analyses were conducted for individual miRNAs and their combinations (Supplementary Table S1). Among single miRNAs, miR-222-3p (AUC = 0.330, *p* = 0.002) and miR-3154 (AUC = 0.289, *p* < 0.001) demonstrated statistically significant but inverse discriminatory power, with AUC values below 0.5. Other single miRNAs, including miR-3945, miR-4534, and miR-4742, exhibited AUC values close to 0.5 and did not show significant diagnostic value. Regarding combinations, the pair miR-222-3p + miR-4534 showed the highest diagnostic potential (AUC = 0.629, *p* = 0.022). A few other two- and three-miRNA combinations approached statistical significance (e.g., AUCs ~0.60), while combinations including miR-3945 consistently yielded AUC values below 0.5, indicating poor diagnostic utility. The best performing three- and four-miRNA combinations showed AUCs of 0.623 (*p* = 0.030) and 0.615 (*p* = 0.043), respectively. However, the five-miRNA combination did not offer improved diagnostic accuracy (AUC = 0.393, *p* = 0.060). Overall, the findings suggest that individual miRNAs, particularly miR-222-3p and miR-3154, and specific combinations such as miR-222-3p + miR-4534 may possess limited but potentially useful inverse discriminatory power in differentiating PDAC from healthy controls.

#### 3.2.2. Correlation of Selected miRNA Expression Levels with Clinical Parameters

The relationship between the expression levels of miR-222-3p, miR-3154, miR-3945, miR-4534, and miR-4742 and various clinicopathologic parameters was investigated using the Kruskal–Wallis test based on 2^−ΔΔCt^ values, and the results are shown in Table 3. Among the clinical variables, a statistically significant difference was observed in miR-222-3p expression levels in relation to alcohol consumption (*p* = 0.033), surgical margin status (*p* = 0.257, not statistically significant but notable difference in medians), locally advanced disease (*p* = 0.035), and distant metastasis (*p* = 0.050). Patients with alcohol consumption or locally advanced disease had higher miR-222-3p expression than their counterparts. miR-3154 expression levels differed significantly by T-stage (*p* = 0.004) and distant metastases (*p* = 0.034) and showed a trend towards significance in surgical margin status (*p* = 0.050) and locally advanced disease (*p* = 0.092). Of note, miR-3154 levels were elevated in advanced T-stage and metastatic cases. For miR-3945, higher expression was associated with distant metastases (*p* = 0.045), although no other parameters reached statistical significance. miR-4534 expression levels varied significantly with lymph node involvement (*p* = 0.023) and locally advanced disease (*p* = 0.013), suggesting a possible role in regional invasion and disease progression. No significant associations were observed for miR-4742 in most comparisons. However, an inverse trend was found for comorbidity (*p* = 0.014), with patients without comorbid conditions having higher miR-4742 expression.

The relationship between the clinical parameters and the combined expression levels of the selected miRNAs was assessed using the Kruskal–Wallis test. Statistically significant differences were observed for the combinations of miR-222-3p + miR-3154 and miR-222-3p + miR-4534 in terms of perineural invasion (*p* = 0.032 and *p* = 0.044, respectively). Regarding tumor stage, significant associations were observed with miR-3154 + miR-4534 (*p* = 0.013) and miR-3154 + miR-4742 (*p* = 0.043). With regard to lymph node positivity, the combinations miR-222-3p + miR-4534 and miR-4534 + miR-4742 were significantly associated (*p* = 0.050). For surgical margin status, significant differences were found for miR-222-3p + miR-3154 (*p* = 0.051), miR-3154 + miR-4534 (*p* = 0.044), miR-3154 + miR-4742 (*p* = 0.014), and miR-4534 + miR-4742 (*p* = 0.030). In addition, alcohol consumption was significantly associated with miR-222-3p + miR-3154 (*p* = 0.036). When tumor stage was evaluated in relation to the triple miRNA combinations, statistically significant differences were observed for miR-222-3p + miR-3154 + miR-4534 (*p* = 0.002), miR-222-3p + miR-3154 + miR-4742 (*p* = 0.003), and miR-3154 + miR-4534 + miR-4742 (*p* = 0.002). A significant association was also found between lymph node status and the combination miR-222-3p + miR-4534 + miR-4742 (*p* = 0.038). As for surgical margin status, significant associations were found for miR-222-3p + miR-3154 + miR-4742 (*p* = 0.030) and miR-3154 + miR-4534 + miR-4742 (*p* = 0.013). Importantly, the quadruple combination of miR-222-3p + miR-3154 + miR-4534 + miR-4742 was significantly associated with surgical margin positivity (*p* = 0.027). In conclusion, both single and multiple miRNA combinations showed significant associations with tumor stage, surgical margin status, and lymph node involvement, suggesting that increased expression levels of these miRNAs may be associated with more advanced or aggressive tumor characteristics.

### 3.3. In Silico Functional Analysis of miRNA Target Genes

**Figure 3:**
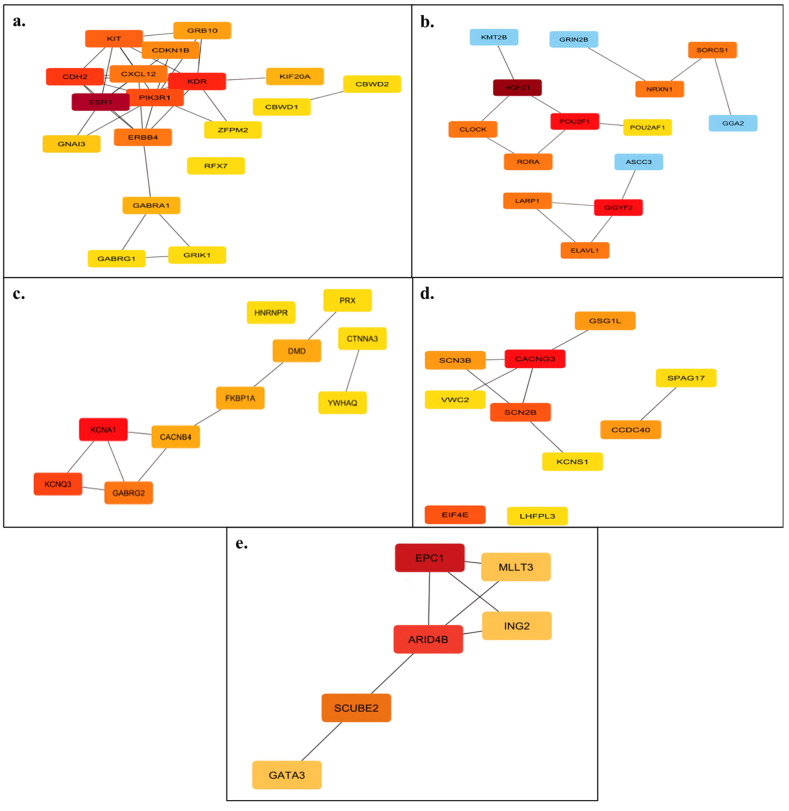
Gene–miRNA interaction networks constructed via Cytoscape for each miRNA. The color gradient represents MCC scores, with red nodes indicating hub genes with higher centrality values. (**a**) miR-222-3p, (**b**) miR-3154, (**c**) miR-3945, (**d**) miR-4534, (**e**) miR-4742.

The gene–miRNA interaction networks constructed using Cytoscape revealed complex node–edge architectures, as illustrated in Figure 3. Each subnetwork corresponds to the predicted targets of individual miRNAs. Centrality analysis was conducted using the CytoHubba plugin, and hub genes were identified based on their Maximal Clique Centrality (MCC) scores. Genes with the highest MCC values were considered central regulatory nodes within their respective networks. Notably, genes such as PIK3R1, CXCL12, and ESR1 (Figure 3a), as well as CLOCK and POU2F1 (Figure 3b), were among the top-ranking hub genes. As a result, ESR1, HCFC1, KCNA1, CACNG3, and EPC1 were identified as the main hub genes targeted by miR-222-3p, miR-3154, miR-3945, miR-4534, and miR-4742, respectively. These results suggest that the corresponding miRNAs may potentially exert regulatory influence over critical genes involved in oncogenic signaling and cellular processes. The presence of interconnected clusters with varying centrality scores further supports the role of these miRNAs in modulating complex gene regulatory networks.

### 3.4. Quantitative Analysis of Targeted Protein Expression Levels

**Figure 4:**
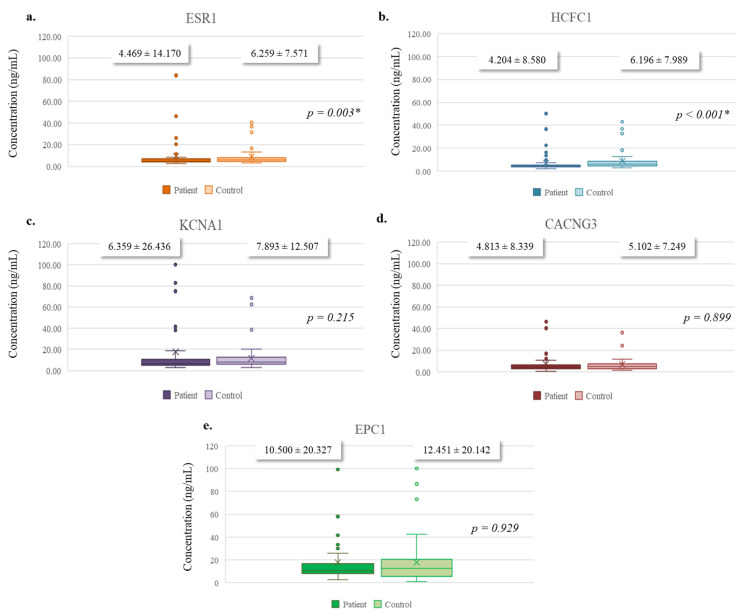
The distribution of levels of the target proteins between PDAC patients and healthy individuals. (**a**) ESR1, (**b**) HCFC1, (**c**) KCNA1, (**d**) CACNG3, and (**e**) EPC1. Differences between the groups were analyzed using the Mann–Whitney U test. Bars represent mean protein concentration, and error bars indicate standard deviation (mean ± SD). * *p* < 0.05 was considered statistically significant.

The serum expression levels of the target proteins were compared between patients with PDAC and healthy individuals using the Mann–Whitney U test. ESR1 expression was significantly lower in patients (mean ± SD: 4.469 ± 14.170) compared to controls (mean ± SD: 6.259 ± 7.571, *p* = 0.003). Similarly, HCFC1 levels were significantly reduced in the patient group (mean ± SD: 4.204 ± 8.580) compared to healthy individuals (mean ± SD: 6.196 ± 7.989, *p* < 0.001). In contrast, no statistically significant differences were observed for KCNA1 (mean ± SD: 6.359 ± 26.436 vs. 7.893 ± 12.507, *p* = 0.215), CACNG3 (mean ± SD: 4.813 ± 8.339 vs. 5.102 ± 7.249, *p* = 0.899), or EPC1 (mean ± SD: 10.500 ± 20.327 vs. 12.451 ± 20.142, *p* = 0.929) between the groups (Figure 4). These results highlight ESR1 and HCFC1 as significantly altered proteins in PDAC, while the remaining proteins showed no statistically significant changes.

**Figure 5:**
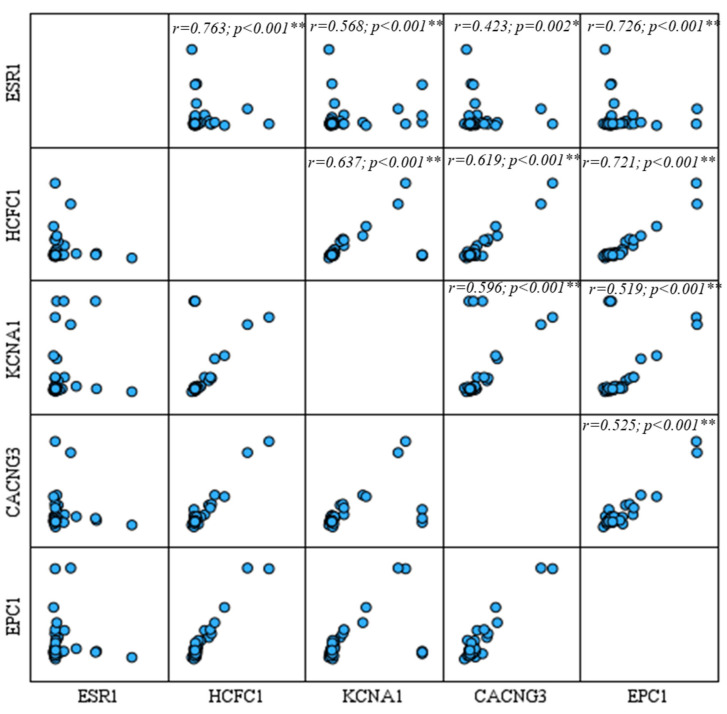
Scatter plot matrix demonstrating the pairwise correlations among protein serum concentration levels of ESR1, HCFC1, KCNA1, CACNG3, and EPC1. The upper triangle of the matrix displays Pearson correlation coefficients (r) with corresponding *p*-values. Significant positive correlations were observed for each miRNA. * *p* < 0.05 and ** *p* < 0.001 were considered statistically significant.

Bivariate Pearson correlation analysis was performed to assess the associations between the expression levels of the target protein in the patient group and visualized through a scatter plot matrix (Figure 5). A strong and statistically significant positive correlation was observed between ESR1 and HCFC1 (r = 0.763; *p* < 0.01). ESR1 also showed significant positive correlations with KCNA1 (r = 0.568; *p* < 0.01), CACNG3 (r = 0.423; *p* = 0.002), and EPC1 (r = 0.726; *p* < 0.01). Similarly, HCFC1 exhibited strong positive correlations with KCNA1 (r = 0.637; *p* < 0.01), CACNG3 (r = 0.619; *p* < 0.01), and EPC1 (r = 0.721; *p* < 0.01). KCNA1 was also significantly positively correlated with both CACNG3 (r = 0.596; *p* < 0.01) and EPC1 (r = 0.519; *p* < 0.01). Furthermore, a significant positive correlation was found between CACNG3 and EPC1 (r = 0.525; *p* < 0.01). These findings indicate that the expression levels of the evaluated genes are significantly interrelated, suggesting that they may be co-regulated or functionally linked in a coordinated manner.

#### 3.4.1. Biomarker Potentials of Target Proteins and Their Combinations

To evaluate the individual diagnostic value of CACNG3, EPC1, ESR1, HCFC1, and KCNA1 proteins in differentiating PDAC patients from healthy controls, ROC analysis was performed using serum protein expression levels (Supplementary Table S2). Among these proteins, HCFC1 demonstrated the lowest diagnostic power, with an AUC of 0.289 (*p* < 0.001), indicating a statistically significant but inverse discriminative capacity. ESR1 also showed a low AUC of 0.330 (*p* = 0.002), suggesting a potential decrease in its expression among patients. In contrast, KCNA1 had an AUC of 0.428 (*p* = 0.223), while CACNG3 (AUC = 0.493; *p* = 0.899) and EPC1 (AUC = 0.505; *p* = 0.930) exhibited AUC values very close to 0.5, with no statistically significant discriminatory power (*p* > 0.05). These findings indicate that CACNG3 and EPC1 do not exhibit meaningful diagnostic capacity in distinguishing PDAC from healthy individuals. Furthermore, when evaluated in combination, none of the protein pairs or multi-marker panels yielded statistically significant improvements in diagnostic performance. The consistently low AUC values observed for HCFC1 and ESR1 may reflect marked downregulation in the patient group, implying potential tumor suppressor roles. Therefore, further studies are warranted to explore the functional relevance and biomarker potential of HCFC1 and ESR1 in PDAC.

#### 3.4.2. Correlation of Targeted Protein Levels with Clinical Parameters

The expression levels of the proteins ESR1, HCFC1, KCNA1, CACNG3, and EPC1 measured by ELISA were evaluated in relation to various clinical and pathological parameters using the Kruskal–Wallis test based on concentrations calculated from the standard curve (ng/mL) (Table 4). It was found that EPC1 expression was significantly higher in cases with lymphocytic invasion (LVI) (*p* = 0.022). Similarly, the expression levels of HCFC1 (*p* = 0.030) and EPC1 (*p* < 0.001) were significantly increased in the presence of LVI. In particular, increased expression of HCFC1 was found in metastatic cases (*p* = 0.023). In addition, both ESR1 and EPC1 expression levels were increased in poorly differentiated tumors, suggesting a possible link between these genes and aggressive tumor phenotypes.

The associations between clinical parameters and binary protein combinations were evaluated using the Kruskal–Wallis test. Among these, only the pairs ESR1 + HCFC1 (*p* = 0.016), ESR1 + CACNG3 (*p* = 0.020), ESR1 + EPC1 (*p* = 0.003), HCFC1 + EPC1 (*p* = 0.003), KCNA1 + EPC1 (*p* = 0.017), and CACNG3 + EPC1 (*p* = 0.003) were found to be statistically significantly associated with lymphovascular invasion. These combinations demonstrated significantly higher mean ranks in LVI-positive cases. In addition, significant associations were observed between lymphovascular invasion and the following triple protein combinations: ESR1 + HCFC1 + CACNG3 (*p* = 0.022), ESR1 + HCFC1 + EPC1 (*p* = 0.004), ESR1 + KCNA1 + EPC1 (*p* = 0.013), ESR1 + CACNG3 + EPC1 (*p* = 0.003), HCFC1 + KCNA1 + EPC1 (*p* = 0.013), HCFC1 + CACNG3 + EPC1 (*p* = 0.003), and KCNA1 + CACNG3 + EPC1 (*p* = 0.020). In these groups as well, LVI-positive samples exhibited significantly higher mean rank values compared to LVI-negative cases. Among the quadruple protein combinations, ESR1 + HCFC1 + KCNA1 + EPC1 (*p* = 0.011), ESR1 + HCFC1 + CACNG3 + EPC1 (*p* = 0.002), ESR1 + KCNA1 + CACNG3 + EPC1 (*p* = 0.013), and HCFC1 + KCNA1 + CACNG3 + EPC1 (*p* = 0.012) were found to be significantly associated with lymphovascular invasion. A significant association was also observed in the quintuple combination ESR1 + HCFC1 + KCNA1 + CACNG3 + EPC1 (*p* = 0.010). In all these combinations, LVI-positive cases showed markedly higher mean rank values compared to those without lymphovascular invasion. Overall, the presence of LVI was shown to be associated with increased levels of the target proteins, particularly in multiple protein combinations.

### 3.5. Correlation and Regression Analyses

**Figure 6:**
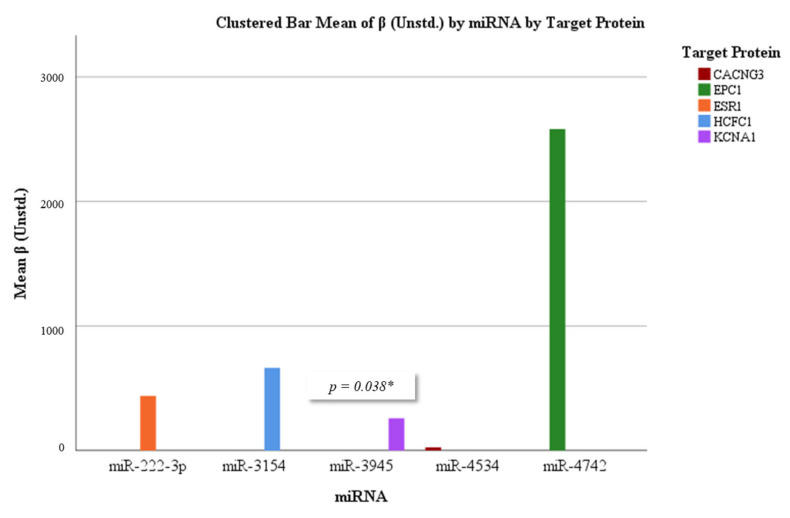
Standardized beta coefficients from linear regression analyses between selected miRNAs and their predicted protein targets. Bars represent the strength and direction of the association. The corresponding target proteins are indicated below each miRNA. Statistically significant associations (*p* < 0.05) are marked with an asterisk.

To further investigate the potential regulatory impact of circulating miRNAs on their predicted protein targets, linear regression models were constructed for each miRNA–protein pair. These findings are detailed in Table 5 and visualized through a regression coefficient plot in Figure 6, which illustrates the direction and strength of associations for each pair. A significant positive correlation was observed between miR-3945 and KCNA1 expression (β = 0.259, *p* = 0.038, R^2^ = 0.094), indicating that increased miR-3945 levels are predictive of increased KCNA1 protein levels. In contrast, analysis of miR-4534 and CACNG3 revealed no significant association (β = 0.025, *p* = 0.968, R^2^ = 0.000), suggesting a lack of predictive influence of this miRNA on CACNG3 expression in the cohort studied. The model examining the association between miR-4742 and EPC1 yielded a marginally non-significant result (β = 2.581, *p* = 0.055, R^2^ = 0.081). Although this association did not reach statistical significance, a potential trend indicating a positive relationship between miR-4742 and EPC1 expression was observed. Overall, these results suggest that although most miRNA–protein pairs did not show significant predictive associations, miR-3945 may serve as a potential positive regulator of KCNA1 expression. Further studies are needed to validate these findings and to investigate the underlying mechanisms of miRNA-mediated regulation in cancer-related signaling pathways.

### 3.6. Survival Analysis

**Figure 7:**
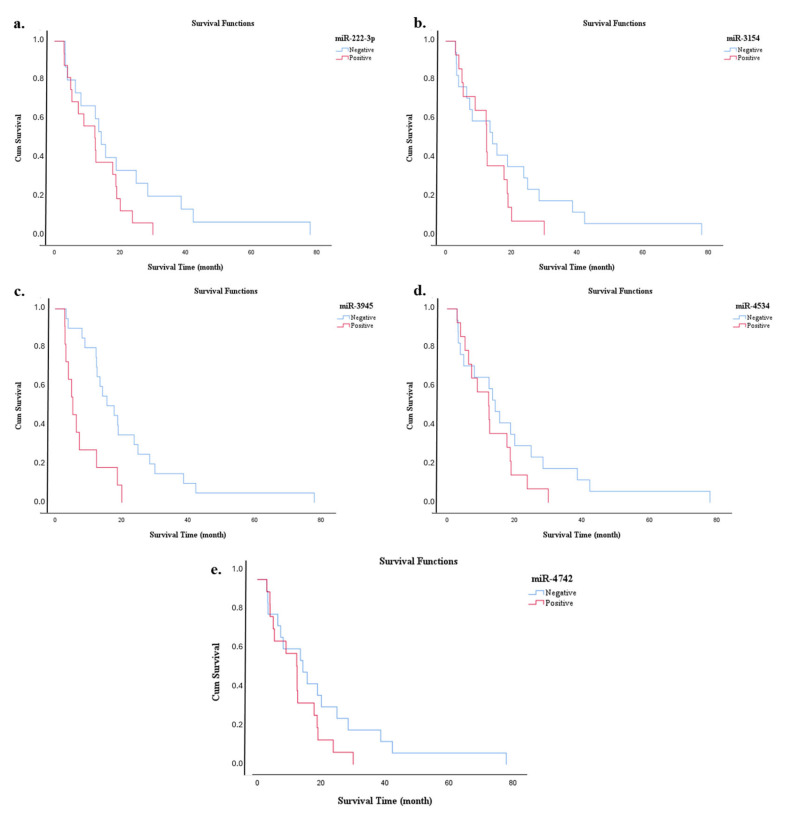
Kaplan–Meier survival curves showing overall survival durations of patients based on the expression levels of selected miRNAs. (**a**) miR-222-3p, (**b**) miR-3154, (**c**) miR-3945, (**d**) miR-4534, and (**e**) miR-4742.

**Figure 8:**
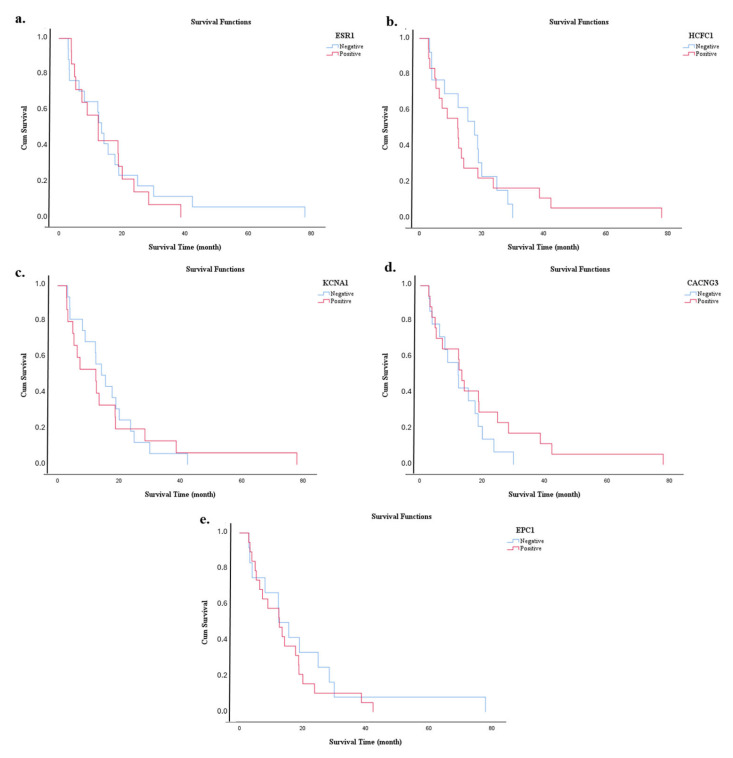
Kaplan–Meier survival curves showing overall survival durations of patients based on the expression levels of target proteins. (**a**) ESR1, (**b**) HCFC1, (**c**) KCNA1, (**d**) CACNG3, and (**e**) EPC1.

A Kaplan–Meier analysis was performed to evaluate the impact of all clinical parameters on overall survival. The median survival time (months), standard error, and 95% confidence intervals are reported for each parameter group (Table 6). The survival differences between the groups were statistically analyzed using the log-rank test. The mean overall survival time of the patients included in the study was 16.6 months, while the median survival time was 13 months, with survival times ranging from 3 to 78 months. Variables such as gender, presence of diabetes, concomitant diseases, smoking and alcohol consumption, family history, anatomical location of the tumor, histological differentiation grade, surgical margin status, lymphovascular and perineural invasion, locally advanced disease, T-stage, lymph node involvement, WHO stage, treatment modality, recurrence, and presence of metastases were individually analyzed for their impact on survival. The effects of miRNA and protein expression levels on survival were also assessed using Kaplan–Meier analysis. For expression-based comparisons, patients were stratified into Low and High expression groups according to the median expression value, with values below the median classified as Low and values above the median as High. The survival curves for miRNAs are shown in Figure 7, and those for protein expression levels are shown in Figure 8. The results of the log-rank test, which summarizes the survival differences based on the expression levels, are shown in Table 6. Of the biomarkers analyzed, only the miR-3945 expression level showed a statistically significant impact on survival (*p* < 0.001). Patients in the group with high miR-3945 expression had a significantly shorter survival time. These results indicate that only the miR-3945 expression level can serve as a significant prognostic indicator for overall survival in this patient cohort.

**Figure 9:**
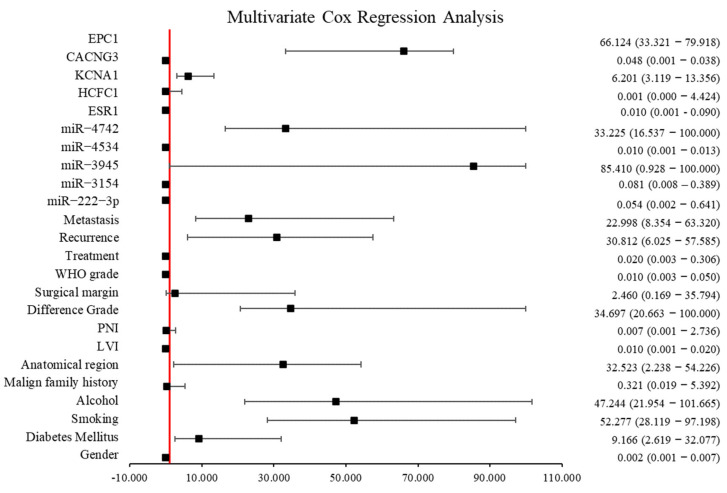
Multivariate Cox regression analysis of the selected miRNAs and their corresponding proteins using clinicopathological variables. The plot illustrates the hazard ratios (Exp(B)) and 95% confidence intervals for each variable. The red line represents the null effect (HR = 1). Values to the right of the line indicate an association with increased risk, whereas those to the left suggest a protective effect.

As a result of multivariate Cox regression analysis, certain clinical and miRNA expression parameters were found to have statistically significant and independent effects on overall survival. In our study, the effects of miRNAs and proteins (as shown in the Figure 9) on disease progression were first evaluated separately. Subsequently, both miRNAs and proteins together with all clinical parameters were simultaneously included in a comprehensive multivariate model. Multivariate regression analysis revealed that several molecular and clinical variables, including EPC1 (OR = 66.124; 95% CI: 33.321–79.918; *p* = 0.006), KCNA1 (OR = 6.201; 95% CI: 3.119–13.356; *p* = 0.017), ESR1 (OR = 0.001; 95% CI: 0.000–0.009; *p* < 0.001), miR-4742 (OR = 33.225; 95% CI: 16.537–100.000; *p* < 0.001), miR-4534 (OR = 0.000; 95% CI: 0.000–0.013; *p* = 0.001), and metastasis (OR = 22.998; 95% CI: 8.354–63.320; *p* < 0.001), were significantly associated with overall survival. In addition, lifestyle-related factors such as smoking (OR = 52.277; 95% CI: 28.119–97.198; *p* = 0.001), alcohol consumption (OR = 47.244; 95% CI: 21.954–101.665; *p* < 0.001), and diabetes (OR = 9.166; 95% CI: 2.619–32.077; *p* = 0.002) were identified as independent risk factors that negatively influence survival. Of note, for WHO grade, miR-3154, and CACNG3, both the lower and upper bounds of the estimated confidence interval were zero, indicating exceptionally strong effects of these variables within the model.

## 4. Discussion

PDACs are associated with poor prognosis due to high mortality rates and frequent diagnosis at advanced stages [18]. The development of chemoresistance by tumor cells, combined with the high incidence of local recurrence and distant metastases, has limited the efficacy of current treatment approaches [19]. This made it necessary to find a novel biomarker not only for treatment but also for early diagnosis and prognostic prediction. In recent years, studies conducted at the molecular level have revealed that gene expression profiles and changes at the protein level may play a critical role in pancreatic carcinogenesis [20]. A comprehensive understanding of both the genetic regulatory mechanisms and the proteomic consequences of these gene products is crucial for advancing the development of diagnostic tools and targeted therapeutic strategies [21]. In this study, the expression levels of miR-222-3p, miR-3154, miR-3945, miR-4534, and miR-4742—which had not been previously well-characterized in relation to PDAC and were identified through microarray experiments—were analyzed in PBMCs obtained from patients with PDAC and healthy individuals. In addition, changes in protein levels of their target genes (ESR1, HCFC1, KCNA1, CACNG3, EPC1) were also evaluated. The findings suggested that these miRNAs and proteins may serve as potential biomarker candidates for both diagnostic and prognostic purposes.

In our study, the expression levels of miR-222-3p, miR-3154, miR-3945, miR-4534, and miR-4742 were analyzed in association with various clinicopathological parameters, leading to several significant findings. In particular, miR-222-3p levels were found to be elevated in patients with a history of alcohol consumption. This observation is consistent with previous studies suggesting that miR-222 may be associated with disease progression in alcohol-related organs such as the liver and pancreas [22,23]. The significant association between miR-3154 and T-stage suggests that this miRNA may influence tumor growth and invasiveness. Although there are few studies on miR-3154, some evidence suggests that it targets genes involved in inflammatory responses and cytoskeletal reorganization. This suggests that its increased expression in advanced tumor stages may be related to increased cell motility and invasion [24]. The increased levels of miR-222-3p observed in perineural invasion suggest a possible role of this miRNA in neurotrophic spread. In the literature, perineural invasion has been associated with poor prognosis in PDAC, and molecular markers interacting with neuronal cells have been reported to play a role in this process [25]. Considering the known association of miR-222-3p with target genes that influence neuronal cell morphology, this finding may be biologically significant. The association of miR-3154, miR-4534, and miR-4742 levels with positive surgical margins suggests a potential link between these miRNAs and local tumor invasion. miR-4534 has been reported to possess oncogenic potential and to enhance cell proliferation in various cancer types [26]. Therefore, the increased expression of miR-4534 observed in association with positive surgical margins may suggest a potential role in enhancing local invasion capacity. The available data on miR-4742 is limited. Notably, miR-4534 has been reported to promote metastatic behavior by targeting the PI3K/AKT signaling pathways [26]. The significant association observed only between tumor stage and miR-3945 suggests that this miRNA may be suppressed in early-stage disease. Although there are currently no direct studies linking miR-3945 specifically to PDAC, it has been reported to be involved in gene expression regulation and associated with epigenetic modulators [27,28,29]. This may imply that its increased expression in advanced stages contributes to genetic instability and tumor progression.

Based on target prediction analyses performed using the miRDB database, miR-222-3p, miR-3154, miR-3945, miR-4534, and miR-4742 were shown to have the potential to target the ESR1, HCFC1, KCNA1, CACNG3, and EPC1 genes, respectively. In gene–miRNA interaction networks constructed using String (SIB—Swiss Institute of Bioinformatics, Lausanne, Switzerland), Cytoscape, and CytoHubba software (v3.10.0, Institute for Systems Biology, Seattle, WA, USA), these genes were identified as hub nodes with high centrality scores. This finding suggested that the corresponding miRNAs may play critical roles in the post-transcriptional regulation of gene expression.

The strong association between miR-222-3p and ESR1 was particularly noteworthy. It has been shown that miR-222-3p functions either as a tumor suppressor or as an oncogene in various cancers [30,31,32] and diabetes mellitus disease [33]. The ESR1 gene plays a key role in hormonal regulation, cell proliferation, and tumor development. In the context of PDAC, changes in the expression of ESR1 have been reported to be associated with disease progression and prognosis [34]. These findings suggest that regulation of ESR1 expression by miR-222-3p may play a role in shaping the biological dynamics of PDAC. Similarly, the regulatory effect of miR-3154 on HCFC1 (Host Cell Factor C1) is also worth mentioning. HCFC1 plays a role in cell cycle regulation and transcriptional control mechanisms [35]. Although data on miR-3154 remain limited, some studies have shown its interactions with important molecular targets in malignancies such as gliomas [24] and liver cancer [36]. Accordingly, miR-3154 may contribute to the pathogenesis of PDAC by modulating cellular proliferation processes via HCFC1. The relationship between miR-3945 and KCNA1 is also of particular interest. KCNA1 encodes voltage-gated potassium channels and is involved in the regulation of membrane potential [37]. Recent studies have shown that KCNA1 can increase metastatic potential and cell motility in certain cancers [38,39]. In this context, miR-3945 may affect the proliferative and invasive abilities of pancreatic cells by suppressing KCNA1 expression. In this study, the potential regulatory effects of miR-4534 on CACNG3 and miR-4742 on EPC1 were also investigated. CACNG3 is a gene involved in calcium channel activity and has been associated with mechanisms such as signal transduction and intracellular calcium homeostasis [40]. Both miR-4534 and CACNG3 have been reported to be involved in cancer-related signaling pathways in various studies [26,41,42]. EPC1, on the other hand, functions in epigenetic regulatory complexes and plays a crucial role in DNA repair and chromatin remodeling [43]. The potential regulatory effect of miR-4742 on EPC1 could influence the genetic stability of cancer cells and contribute to mechanisms of therapy resistance. Overall, the findings obtained from the constructed gene–miRNA interaction networks suggest that the expression levels of target proteins can be regulated not only by genetic mutations but also post-transcriptionally via miRNAs.

Spearman correlation analyses showed significant and positive correlations between all evaluated proteins. In particular, the high correlation coefficients observed between ESR1 and HCFC1 and between HCFC1 and EPC1 suggest that the expression of these genes may be co-regulated. These results suggest that transcriptional or epigenetic mechanisms may influence these genes via common regulatory pathways. Similarly, the moderate but significant correlations between KCNA1, CACNG3, and EPC1 point toward their potential roles in cellular signaling or membrane potential regulation. These co-expression patterns further highlight the complex molecular network underlying PDAC progression and support the use of multi-marker panels in both diagnostic and prognostic contexts.

In patients with PDAC, the serum levels of the proteins ESR1, HCFC1, KCNA1, CACNG3, and EPC1 were measured using the ELISA method, and their correlation with various clinicopathological parameters was investigated. In univariate analyses, it was found that the expression levels of ESR1, HCFC1, and EPC1 were significantly increased in the presence of lymphatic invasion. In addition, HCFC1 expression was observed to be significantly increased in patients with metastatic disease. These results suggest that the above proteins may serve as potential biomarkers for aggressive tumor biology. Biologically noteworthy but not statistically significant findings included that protein levels of KCNA1 and CACNG3 were higher in tumors localized to the posterior part of the pancreas. This trend could indicate a shift in molecular profiles depending on the anatomical location of the tumor. In addition, the increased expression of ESR1 and EPC1 in poorly differentiated tumors supports a possible link between these genes and aggressive tumor phenotypes.

The linear regression analyses performed in this study shed light on the possible regulatory interactions between circulating miRNAs and their predicted protein targets in serum. While most miRNA–protein pairs did not show statistically significant associations, the observed positive correlation between miR-3945 and KCNA1 suggests that miR-3945 could contribute to the regulation of KCNA1 expression and thus potentially influence tumor- or immune-related processes. In addition, the marginal association between miR-4742 and EPC1 could indicate a weak but biologically relevant interaction that could become more apparent with a larger sample size or in other biological contexts. On the other hand, the lack of significance in pairs such as miR-222-3p/ESR1 and miR-4534/CACNG3 could be due to limitations imposed by post-transcriptional regulatory complexity, redundancy among miRNAs, or insufficient statistical power. These results highlight the complexity of miRNA-based regulation and emphasize the importance of performing further functional studies to elucidate the underlying mechanisms.

The prognostic values of certain miRNA and protein expression levels, along with various clinical parameters that could potentially influence survival in patients with PDAC, were evaluated using Kaplan–Meier survival analysis in this study. The findings indicated that the mean overall survival duration was 16.6 months, while the median survival was 13 months. The wide range of survival times (3–78 months) reflected the heterogeneity of the patient group and the variability in the biological behavior of the disease. When assessed in terms of clinical parameters, demographic and clinical factors such as sex, presence of diabetes, comorbidities, smoking and alcohol use, and family history were not found to have a statistically significant effect on survival [44]. A noticeable difference in survival was observed among patients, depending on the anatomical location of the tumor. For example, tumors in the tail of the pancreas were associated with a shorter survival time than tumors in other regions. This could be due to the fact that tumors in the tail region of the pancreas often show symptoms later, leading to diagnosis at more advanced stages of disease [45]. Patients who received chemotherapy or chemoradiotherapy survived significantly longer than patients who received no treatment, emphasizing the critical role of therapeutic intervention in disease progression. In particular, treatment protocols that include radiotherapy have been shown to provide a survival benefit, as previous studies have demonstrated [46]. At the molecular level, the influence of miRNA and protein expression on survival has been investigated. Of these, only the expression level of miR-3945 showed a statistically significant correlation with overall survival. Patients with high miR-3945 expression had significantly shorter survival, suggesting that miR-3945 may serve as a potential prognostic biomarker in PDAC. Although there is limited specific data on miR-3945 in the literature, other miRNAs with similar mechanisms—such as miR-21 and miR-155—have been associated with aggressive tumor behavior and poor prognosis [47]. Further in-depth molecular studies are needed to elucidate how miR-3945 affects cellular proliferation, invasion, and apoptosis pathways, which would be essential to validate this preliminary finding. No statistically significant differences in survival were observed for the other miRNAs (miR-222-3p, miR-3154, miR-4534, miR-4742) and proteins (ESR1, HCFC1, KCNA1, CACNG3, EPC1) examined. This may indicate that these biomarkers have limited prognostic value or that the sample size used in this study did not have sufficient statistical power to detect such associations. Although miR-222-3p has been shown to play a prognostic role in hepatocellular carcinoma, the lack of a similar effect in other tumor types such as PDAC may be due to underlying biological differences [31,32]. These findings emphasize that the influence of clinical and molecular parameters on the survival of PDAC patients is multifactorial and highly patient-specific. The association between high miR-3945 expression and poor prognosis underpins the potential of miR-3945 as a prognostic biomarker at an advanced level. Validation of these findings through large-scale, prospective studies in the future will improve our understanding of disease biology and contribute to the development of personalized treatment strategies. In the multivariate analysis, which included both miRNAs and proteins, several molecular markers—including EPC1, ESR1, and KCNA1—and lifestyle factors such as smoking and alcohol consumption were found to have independent and significant effects on survival. These findings highlight the combined influence of genetic/molecular and environmental factors on disease prognosis and emphasize the need to consider such interactions in clinical decision-making. The strong associations observed between modifiable risk factors—such as smoking and alcohol consumption—and unfavorable survival outcomes also underscore the importance of public health interventions [48]. In addition, the presence of confidence intervals on the edge of zero for variables such as WHO grade and CACNG3 suggests that these factors may have strong effects within the model, but such results should be interpreted with caution. The potential influence of these outlier variables emphasizes the need for confirmation in future analyses with larger, more representative sample sizes. The overall significance of the multivariate model was confirmed by likelihood ratio tests, suggesting that the included variables jointly contribute to the predictive performance of the model. These results suggest that the identified molecular and clinical parameters could serve as potential prognostic markers in patients with PDAC. Nevertheless, further validation in larger, independent cohorts is warranted to confirm the robustness of these associations.

The strengths of the study include the analysis of both mRNA and protein expression levels, the corroboration of the results by functional in silico analyses, and the integration of the molecular data with clinical parameters such as survival. This study has several limitations that should be noted. First, the sample size was relatively small, which may have limited the statistical power to detect more subtle relationships, particularly in subgroup analyses. The limited number of cases was primarily due to the strict inclusion criteria—such as the restriction to untreated patients—and the focus on a specific histologic subtype of PDAC (pancreatic ductal adenocarcinoma), which naturally limited the eligible study population. Secondly, peripheral blood lymphocytes were used instead of tumor tissue for miRNA and protein expression profiling. This approach was chosen because, in many cases, only limited biopsy material was available, and preservation of samples for the necessary pathological evaluation was a priority. Therefore, the use of blood material may not fully reflect the tumor microenvironment. Third, the cross-sectional design of the study prevents any causal inferences about the observed associations. These limitations underscore the need for future large-scale, prospective, and mechanistic studies using tumor tissue and broader patient cohorts to validate and extend our findings.

## 5. Conclusions

This study suggests that certain miRNAs and their corresponding target proteins may be associated with potential diagnostic and prognostic significance in pancreatic cancer, although further validation is required. The downregulation of miR-222-3p and miR-3154 in patient samples and their associations with clinical features such as alcohol consumption and tumor stage underscore their potential importance in characterizing the disease. Importantly, increased expression of miR-3945 was associated with shorter survival, suggesting its possible role as a prognostic marker. Furthermore, the positive correlation between miR-3945 and KCNA1 expression suggests that miR-3945 may influence tumor- or immune-related signaling pathways by modulating KCNA1. At the protein level, significant associations with lymphatic invasion and survival were observed, highlighting the biological impact of the selected targets. A major strength of this work lies in its integrated approach—combining molecular data at both RNA and protein levels, supporting these findings with computational predictions and correlating them with clinically meaningful outcomes such as survival. Further validation in larger, well-characterized patient cohorts, supported by functional studies, is needed to better understand the mechanistic role of these candidate biomarkers and their potential translational applications in PDAC.

## Figures and Tables

**Table 1:** miRNA primer and stem-loop sequences used in the study.

miRNA	Primer (Forward) Sequence (5′→3′)	Stem-Loop Sequence (5′→3′)
hsa-miR-222-3p (F)	AACGCCATTATCACACTAAATA	GAAAGAAGGCGAGGAGCAGATCGAGGAAGAAGACGGAAGAATGTGCGTCTCGCCTTCTTTCTATTTAGT
hsa-miR-3154 (F)	CAGAAGGGGAGTTGGGAGCAGA	GAAAGAAGGCGAGGAGCAGATCGAGGAAGAAGACGGAAGAATGTGCGTCTCGCCTTCTTTCTCTGCTCC
hsa-miR-3945 (F)	AGGGCATAGGAGAGGGTTGATAT	GAAAGAAGGCGAGGAGCAGATCGAGGAAGAAGACGGAAGAATGTGCGTCTCGCCTTCTTTCATATCAAC
hsa-miR-4534 (F)	GGATGGAGGAGGGGTCT	GAAAGAAGGCGAGGAGCAGATCGAGGAAGAAGACGGAAGAATGTGCGTCTCGCCTTCTTTCAGACCCCT
hsa-miR-4742-3p (F)	TCTGTATTCTCCTTTGCCTGCAG	GAAAGAAGGCGAGGAGCAGATCGAGGAAGAAGACGGAAGAATGTGCGTCTCGCCTTCTTTCCTGCAGGC
Universal Reverse (UR)	CGAGGAAGAAGACGGAAGAAT	
U6	GCTTCGGCAGCACATATACTAAAAT	

F: forward.

**Table 2:** Demographic and clinicopathological characteristics of patients with pancreatic ductal adenocarcinoma.

Clinical Parameters		N	%
Age—median (minimum–maximum):		53.5 (34–90)	
Gender	Female	22	47.8
	Male	24	52.2
Smoking	No	20	43.5
	Yes	26	56.5
Alcohol	No	36	78.3
	Yes	10	21.7
Family history of malignancy	No	21	45.7
	Yes	25	54.3
Diabetes mellitus	No	26	56.5
	Yes	20	43.5
Comorbidity	No	12	26.1
	Yes	34	73.9
Anatomical involvement	Head	28	60.9
	Corpus	10	21.7
	Tail	8	17.4
Degree of differentiation	High		0
	Medium	36	78.3
	Low	10	21.7
Surgical margin	Negative	24	52.2
	Positive	22	47.8
Lymphovascular invasion	No	8	17.4
	Yes	38	82.6
Perineural invasion	No	13	28.3
	Yes	33	71.7
T-stage	II	17	37.0
	III	21	45.7
	IV	8	17.4
Lymph node involvement (N)	No	11	23.9
	Yes	35	76.1
Metastasis (M)	No	25	54.3
	Yes	21	46.7
WHO stage	Ib	5	10.9
	IIa	4	8.7
	IIb	11	23.9
	III	5	10.9
	IV	21	45.7
Locally advanced status	No	9	19.6
	Yes	37	80.4
Treatment	No	1	2.2
	CT	27	58.7
	CRT	18	39.1
Recurrence	No	39	84.8
	Yes	7	15.2
Distant metastasis	No	33	71.7
	Yes	13	28.3
Current status	Alive	15	32.6
	Ex	31	67.4

**Table 3:** Evaluation of the association between the expression levels of miR-222-3p, miR-3154, miR-3945, miR-4534, and miR-4742 (reported as median 2^−ΔΔCt^ values) and various clinicopathological parameters using the Kruskal–Wallis test.

Clinic Parameters			miR-222-3p		miR-3154		miR-3945		miR-4534		miR-4742	
		N	Median	*p*	Median	*p*	Median	*p*	Median	*p*	Median	*p*
**Gender**	Female	22	23.20	0.886	24.27	0.709	26.05	0.218	22.82	0.741	21.18	0.262
	Male	24	23.77		22.79		21.17		24.13		25.63	
**Smoking**	No	20	23.00	0.825	25.85	0.298	25.15	0.465	25.60	0.352	24.10	0.790
	Yes	26	23.88		21.69		22.23		21.88		23.04	
**Alcohol**	No	36	21.28	0.033	22.71	0.448	22.33	0.263	21.97	0.143	22.08	0.164
	Yes	10	31.50		26.35		27.70		29.00		28.60	
**Family history of malignancy**	No	21	24.57	0.620	24.29	0.716	24.48	0.651	26.55	0.158	26.86	0.120
	Yes	25	22.60		22.84		22.68		20.94		20.68	
**Diabetes mellitus**	No	26	21.81	0.330	22.40	0.528	23.77	0.877	22.85	0.706	23.38	0.947
	Yes	20	25.70		24.93		23.15		24.35		23.65	
**Comorbidity**	No	12	26.75	0.329	26.83	0.317	25.54	0.540	26.21	0.416	31.67	0.014
	Yes	34	22.35		22.32		22.78		22.54		20.62	
**Anatomical involvement**	Head	28	21.61	0.255	20.46	0.083	21.82	0.594	21.45	0.070	22.07	0.268
	Corpus	10	29.75		31.40		26.15		32.05		29.60	
	Tail	8	22.31		24.25		26.06		20.00		20.88	
**Degree of** **differentiation**	Medium	36	23.32	0.863	22.68	0.432	23.85	0.739	23.40	0.926	23.08	0.690
	Low	10	24.15		26.45		22.25		23.85		25.00	
**Surgical margin**	No	24	21.35	0.257	19.81	0.050	20.67	0.135	20.33	0.095	19.46	0.033
	Yes	22	25.84		27.52		26.59		26.95		27.91	
**LVI**	No	8	23.25	0.963	28.13	0.291	26.06	0.578	26.69	0.470	24.25	0.875
	Yes	38	23.55		22.53		22.96		22.83		23.34	
**PNI**	No	13	17.46	0.055	19.88	0.252	23.19	0.922	22.04	0.643	22.38	0.724
	Yes	33	25.88		24.92		23.62		24.08		23.94	
**T-stage**	II	17	22.62	0.155	19.35	0.004	20.26	0.434	20.06	0.142	20.76	0.198
	III	21	21.14		21.67		25.86		23.29		22.86	
	IV	8	31.56		37.13		24.19		31.38		31.00	
**Lymph Node**	Negative	11	29.82	0.073	28.73	0.139	26.18	0.447	31.55	0.023	28.55	0.153
	Positive	35	21.51		21.86		22.66		20.97		21.91	
**M stage**	No	25	24.08	0.749	23.90	0.825	25.66	0.234	23.28	0.903	22.72	0.667
	Yes	21	22.81		23.02		20.93		23.76		24.43	
**WHO grade**	Ib	5	35.80	0.120	27.20	0.435	24.40	0.210	31.00	0.079	31.40	0.315
	IIa	4	26.63		34.13		37.00		35.50		29.00	
	IIb	11	17.09		19.68		25.50		16.45		18.09	
	III	5	25.70		21.70		18.20		20.80		19.20	
	IV	21	22.81		23.02		20.93		23.76		24.43	
**Locally advanced status**	No	9	31.72	0.035	30.28	0.092	30.00	0.106	33.00	0.013	30.33	0.090
	Yes	37	21.50		21.85		21.92		21.19		21.84	
**Treatment**	Yok	1	11.00	0.721	4.00	0.277	13.00	0.356	22.00		13.00	
	CT	27	23.70		22.74		21.61		21.72		24.67	
	CRT	18	23.89		25.72		26.92		26.25		22.33	
**Recurrence**	No	39	23.96	0.590	23.32	0.834	24.68	0.165	23.22	0.742	23.87	0.682
	Yes	7	20.93		24.50		16.93		25.07		21.43	
**Distant metastasis**	No	33	25.91	0.050	26.14	0.034	25.98	0.045	24.88	0.267	25.09	0.200
	Yes	13	17.38		16.81		17.19		20.00		19.46	

LVI: lymphovascular invasion; PNI: perineural invasion; T: tumorCT: chemotherapy; CRT: chemoradiotherapy; *p*: significance value.

**Table 4:** Evaluation of the association between serum protein levels of ESR1, HCFC1, KCNA1, CACNG3, and EPC1 (reported as median concentrations, ng/mL, calculated from the ELISA standard curve) and various clinicopathological parameters using the Kruskal–Wallis test.

Clinic Parameters			ESR1		HCFC1		KCNA1		CACNG3		EPC1	
		N	Median	*p*	Median	*p*	Median	*p*	Median	*p*	Median	*p*
**Gender**	Female	22	24.27	0.567	23.71	0.404	23.46	0.341	20.54	0.021	24.08	0.509
	Male	24	26.63		27.15		27.38		30.08		26.81	
**Smoking**	No	20	24.66	0.718	26.00	0.830	24.59	0.696	22.32	0.171	22.14	0.148
	Yes	26	26.16		25.11		26.21		28.00		28.14	
**Alcohol**	No	36	24.44	0.331	25.10	0.717	23.85	0.131	24.92	0.598	24.44	0.331
	Yes	10	29.27		26.91		31.36		27.55		29.27	
**Family history of malignancy**	No	21	25.04	0.838	27.96	0.271	24.52	0.661	27.30	0.419	26.74	0.579
	Yes	25	25.89		23.41		26.33		23.96		24.44	
**Diabetes mellitus**	No	26	26.04	0.769	26.18	0.710	24.14	0.468	25.39	0.953	25.93	0.815
	Yes	20	24.82		24.64		27.23		25.64		24.95	
**Comorbidity**	No	12	26.08	0.438	18.71	0.150	17.92	0.094	22.00	0.652	20.50	0.368
	Yes	34	22.59		25.19		25.47		24.03		24.56	
**Anatomical involvement**	Head	28	23.55	0.472	23.05	0.257	23.18	0.277	21.14	0.106	22.57	0.402
	Corpus	10	19.95		19.60		19.50		22.90		21.50	
	Tail	8	27.75		29.94		29.63		32.50		29.25	
**Degree of differentiation**	Medium	36	22.49	0.331	22.50	0.338	23.97	0.651	22.89	0.558	22.53	0.351
	Low	10	27.15		27.10		21.80		25.70		27.00	
**Surgical margin**	No	24	20.19	0.080	25.75	0.235	25.04	0.416	25.42	0.312	26.33	0.135
	Yes	22	27.11		21.05		21.82		21.41		20.41	
**LVI**	No	8	16.50	0.020	17.09	0.030	21.64	0.320	20.55	0.202	13.64	0.002
	Yes	38	28.04		27.87		26.59		26.90		28.85	
**PNI**	No	13	21.38	0.503	22.38	0.735	25.73	0.485	26.15	0.408	21.54	0.549
	Yes	33	24.33		23.94		22.62		22.45		24.27	
**T-stage**	II	17	18.29	0.120	27.82	0.212	24.12	0.153	25.65	0.494	25.71	0.695
	III	21	27.29		21.81		26.10		23.55		22.24	
	IV	8	24.63		18.75		15.38		18.81		22.13	
**Lymph node**	Negative	11	18.55	0.160	22.18	0.709	21.50	0.571	20.73	0.432	21.55	0.580
	Positive	35	25.06		23.91		24.13		24.37		24.11	
**M stage**	No	25	24.44	0.604	19.38	0.023	23.18	0.860	20.38	0.085	21.64	0.305
	Yes	21	22.38		28.40		23.88		27.21		25.71	
**WHO grade**	Ib	5	13.60	0.107	29.60	0.052	22.90	0.644	24.20	0.446	29.20	0.569
	IIa	4	24.00		19.00		24.75		16.50		19.50	
	IIb	11	31.73		15.59		26.45		20.77		19.64	
	III	5	19.60		17.80		15.00		18.80		20.20	
	IV	21	22.38		28.40		23.88		27.21		25.71	
**Locally advanced status**	No	9	18.22	0.198	24.89	0.738	23.72	0.958	20.78	0.508	24.89	0.741
	Yes	37	24.78		23.16		23.45		24.16		23.16	
**Treatment**	Yok	1	7.00	0.478	29.00	0.343	26.00	0.984	34.00	0.138	3.00	0.086
	CT	27	23.07		25.78		23.26		26.02		26.15	
	CRT	18	25.06		19.78		23.72		19.14		20.67	
**Recurrence**	No	39	25.64	0.867	25.49	0.989	25.84	0.685	25.40	0.900	25.33	0.834
	Yes	7	24.64		25.57		23.43		26.14		26.57	
**Distant metastasis**	No	33	25.96	0.721	24.75	0.560	24.14	0.290	23.58	0.136	24.83	0.604
	Yes	13	24.32		27.43		29.00		30.43		27.21	

LVI: lymphovascular invasion; PNI: perineural invasion; T: tumorCT: chemotherapy; CRT: chemoradiotherapy; *p*: significance value.

**Table 5:** Summary of linear regression analyses between miRNAs and their predicted protein targets.

miRNA	Target Protein	β (Unstd.)	*p*-Value	R^2^	Significant
miR-222-3p	ESR1	0.438	0.588	0.007	No
miR-3154	HCFC1	0.663	0.397	0.016	No
miR-3945	KCNA1	0.259	0.038	0.094	Yes
miR-4534	CACNG3	0.025	0.968	0.000	No
miR-4742	EPC1	2.581	0.055	0.081	Borderline

**Table 6:** Results of the log-rank test for overall survival according to clinical parameters and biomarker expression levels (patients were stratified into Low and High expression groups based on the median expression value).

Marker	Expression Level	Estimate (Months)	Std. Error	95% CI		Log Rank (Mantel–Cox)
				Lower	Upper	
**miR-222-3p**	Low	14.267	2.018	10.311	18.222	0.139
	High	12.333	3.500	5.473	19.193	
	Overall	12.633	1.076	10.525	14.742	
**miR-3154**	Low	14.267	5.145	4.183	24.351	0.211
	High	12.467	0.123	12.225	12.709	
	Overall	12.633	1.076	10.525	14.742	
**miR-3945**	Low	15.600	3.913	7.930	23.270	0.001 *
	High	5.333	1.321	2.744	7.923	
	Overall	12.633	1.076	10.525	14.742	
**miR-4534**	Low	14.267	2.149	10.054	18.480	0.208
	High	12.333	3.274	5.916	18.750	
	Overall	12.633	1.076	10.525	14.742	
**miR-4742**	Low	14.267	2.100	10.151	18.383	0.130
	High	12.467	2.214	8.128	16.805	
	Overall	12.633	1.076	10.525	14.742	
**ESR1**	Low	13.500	1.326	10.901	16.099	0.660
	High	12.467	3.240	6.116	18.818	
	Overall	12.633	1.076	10.525	14.742	
**HCFC1**	Low	17.767	3.755	10.408	25.126	0.943
	High	12.333	3.712	5.057	19.609	
	Overall	12.633	1.076	10.525	14.742	
**KCNA1**	Low	14.267	3.133	8.125	20.408	0.823
	High	12.467	4.015	4.598	20.335	
	Overall	12.633	1.076	10.525	14.742	
**CACNG3**	Low	12.333	3.274	5.916	18.750	0.240
	High	13.500	1.235	11.080	15.920	
	Overall	12.633	1.076	10.525	14.742	
**EPC1**	Low	12.467	2.829	6.922	18.012	0.432
	High	12.633	3.289	6.187	19.079	
	Overall	12.633	1.076	10.525	14.742	

* Statistically significant associations (*p* < 0.05) are marked with an asterisk.

## Data Availability

The dataset used during the current study is available from the corresponding author upon reasonable request.

## Supplementary Materials

The following supporting information can be downloaded at https://www.mdpi.com/article/10.3390/diagnostics15192479/s1: Table S1: Diagnostic performance of individual and combined miRNAs in distinguishing PDAC patients from healthy controls, based on ROC curve analysis. Table S2: Diagnostic performance of individual and combined proteins in distinguishing PDAC patients from healthy controls, based on ROC curve analysis.
