# Supplementary material for: A Pilot Study of Exploring miRNA–Protein Interaction Networks in Pancreatic Ductal Adenocarcinoma Patients: Implications for Diagnosis and Prognosis
Source: Diagnostics (Basel). 2025 Sep 27;15(19):2479. doi: 10.3390/diagnostics15192479 (PMC12523441; doi:10.3390/diagnostics15192479)
Supplement: Supplementary file 1 [file diagnostics-15-02479-s001.zip › diagnostics-3801549-supplementary.pdf]

Supplementary Table S1. Diagnostic performance of individual and combined miRNAs in distinguishing PDAC patients from healthy controls, based on ROC curve analysis.

Test Result Variable(s):	Area	Std. Error[a]	Asymptotic Sig.[b]	Asymptotic 95% CI Lower Bound	Asymptotic 95% CI Upper Bound
miR-222-3p	0.33	0.056	0.002	0.22	0.44
miR-3154	0.289	0.055	<0.001	0.182	0.396
miR-3945	0.428	0.059	0.223	0.312	0.544
miR-4534	0.493	0.058	0.899	0.378	0.607
miR-4742	0.505	0.059	0.93	0.389	0.622
miR-222-3p + miR-3154	0.605	0.058	0.069	0.492	0.718
miR-222-3p + miR-3945	0.368	0.056	0.018	0.258	0.477
miR-222-3p + miR-4534	0.629	0.056	0.022	0.519	0.74
miR-222-3p + miR-4742	0.598	0.057	0.088	0.485	0.71
miR-3154 + miR-3945	0.347	0.055	0.005	0.24	0.454
miR-3154 + miR-4534	0.607	0.057	0.06	0.495	0.719
miR-3154 + miR-4742	0.609	0.057	0.054	0.498	0.72
miR3945 + miR-4534	0.362	0.056	0.013	0.253	0.471
miR-3945 + miR-4742	0.354	0.055	0.008	0.246	0.462
miR-4534 + miR-4742	0.609	0.057	0.057	0.497	0.722
miR-222-3p + miR +3154 +miR-3945	0.378	0.057	0.031	0.267	0.489
miR-222-3p + miR-3154 + miR-4534	0.623	0.057	0.03	0.512	0.734
miR-222-3p + miR-3154 + miR-4742	0.601	0.057	0.078	0.489	0.713
miR-222-3p + miR-3945 + miR-4534	0.389	0.057	0.051	0.277	0.501
miR-222-3p + miR-3945 + miR-4742	0.382	0.057	0.037	0.27	0.493
miR-222-3p + miR-4534 + miR-4742	0.606	0.057	0.064	0.494	0.719
miR-3154 + miR-3945 + miR-4534	0.368	0.057	0.018	0.259	0.478
miR-3154 + miR-3945 + miR-4742	0.363	0.056	0.014	0.254	0.472
miR-3154 + miR-4534 + miR-4742	0.606	0.056	0.063	0.494	0.718
miR-3945 + miR-4534 + miR-4742	0.377	0.057	0.029	0.267	0.488
miR-222-3p + miR-3154 + miR-3945 + miR-4534	0.389	0.057	0.052	0.277	0.501
miR-222-3p + miR-3154 + miR-3945 + miR-4742	0.384	0.057	0.042	0.273	0.496
miR-222-3p + miR-3154 + miR-4534 + miR-4742	0.615	0.057	0.043	0.504	0.727
miR-222-3p + miR-3945 + miR-4534 + miR-4742	0.392	0.057	0.059	0.28	0.504
miR-3154 + miR-3945 + miR-4534 + miR-4742	0.378	0.056	0.03	0.268	0.488
miR-222-3p+miR-3154 + miR-3945 + miR-4534 + miR-4742	0.393	0.057	0.060	0.281	0.505

a. Under the nonparametric assumption

b. Null hypothesis: true area = 0.5

Supplementary Table S2. Diagnostic performance of individual and combined protein in distinguishing PDAC patients from healthy controls, based on ROC curve analysis.

Test Result Variable(s):	Area	Std. Error[a]	Asymptotic Sig.[b]	Asymptotic 95% CI	
				Lower Bound	Upper Bound
ESR1	0.330	0.056	0.002	0.220	0.440
HCFC1	0.289	0.055	<0.001	0.182	0.396
KCNA1	0.428	0.059	0.223	0.312	0.544
CACNG3	0.493	0.058	0.899	0.378	0.607
EPC1	0.505	0.059	0.930	0.389	0.622
ESR1+KCNA1	0.402	0.059	0.099	0.286	0.518
ESR1+CACNG3	0.429	0.058	0.225	0.315	0.544
ESR1+EPC1	0.472	0.059	0.632	0.356	0.588
HCFC1+KCNA1	0.374	0.059	0.032	0.259	0.489
HCFC1+CACNG3	0.379	0.058	0.036	0.266	0.492
HCFC1+EPC1	0.456	0.059	0.457	0.340	0.572
KCNA1+CACNG3	0.457	0.059	0.464	0.341	0.572
KCNA1+EPC1	0.499	0.059	0.989	0.383	0.616
CACNG3+EPC1	0.508	0.059	0.898	0.392	0.624
ESR1+HCFC1+KCNA1	0.377	0.059	0.037	0.261	0.493
ESR1+HCFC1+CACNG3	0.378	0.058	0.035	0.264	0.492
ESR1+HCFC1+EPC1	0.433	0.059	0.258	0.317	0.549
ESR1+KCNA1+CACNG3	0.434	0.059	0.269	0.318	0.551
ESR1+KCNA1+EPC1	0.481	0.059	0.747	0.364	0.597
ESR1+CACNG3+EPC1	0.479	0.059	0.720	0.363	0.595
HCFC1+KCNA1+CACNG3	0.412	0.059	0.133	0.296	0.527
HCFC1+KCNA1+EPC1	0.465	0.060	0.554	0.348	0.581
HCFC1+CACNG3+EPC1	0.460	0.059	0.493	0.344	0.575
KCNA1+CACNG3+EPC1	0.500	0.059	1.000	0.384	0.616
ESR1+HCFC1+ KCNA1+CACNG3	0.412	0.059	0.135	0.296	0.528
ESR1+ HCFC1+ KCNA1+ EPC1	0.455	0.059	0.449	0.338	0.572
ESR1+HCFC1+CACNG3+ EPC1	0.438	0.060	0.293	0.321	0.554
ESR1+KCNA1+CACNG3+EPC1	0.484	0.059	0.783	0.367	0.600
HCFC1+KCNA1+CACNG3+EPC1	0.471	0.056	0.624	0.354	0.587
ESR1+HCFC1+KCNA1+CACNG3+EPC1	0.460	0.055	0.499	0.342	0.577

a. Under the nonparametric assumption

b. Null hypothesis: true area = 0.5
